# The Scope and Limits of Iconic Prosody: Head Angle Predicts *f*0 Changes While Object Size Effects Are Absent

**DOI:** 10.1162/OPMI.a.251

**Published:** 2025-12-15

**Authors:** Aleksandra Ćwiek, Susanne Fuchs

**Affiliations:** Leibniz-Centre General Linguistics, Berlin, Germany; Centre for Language Evolution Studies, Nicolaus Copernicus University, Toruń, Poland

**Keywords:** iconicity, prosody, Bayesian modeling, fundamental frequency, sensorimotor, crossmodal correspondence

## Abstract

The relation between the fundamental frequency of the voice (*f*0) and vertical space has been shown in previous studies; however, the underlying mechanisms are less clear. This study investigates the relationship between head angle and *f*0 in iconic prosody, along with the influence of object size on lip opening and formant frequencies. In the experiment, participants pointed to objects of two different sizes and in various vertical positions while saying the words “piff” or “paff,” which induced vertical head position change. Head angle emerged as a reliable predictor of *f*0, with a larger angle increasing the *f*0. This effect was consistent despite individual variations in head movement. While the vertical position of the object also showed a reliable effect on *f*0, head angle substantially outperformed it as a predictor, suggesting that head angle represents the primary physiological mechanism predicting *f*0 changes. Conversely, object size did not predict either lip opening or formant dispersion. Lip opening and formant dispersion were purely indexical, tracking vowel-specific articulatory configurations rather than external object properties. These findings underscore the role of head position in modulating *f*0 through direct physiological coupling, potentially underpinning iconic prosody, while revealing the limits of size-related iconicity in parameters constrained by phonemic requirements.

## INTRODUCTION

Iconicity refers to “a signal in any modality or medium [whose] production and/or perception involves a sense of resemblance between at least some aspect of its form and at least some aspect of its meaning” (Winter et al., in press). In recent years, it has been shown to be widespread across various linguistic levels (Ćwiek, 2022). One area where this phenomenon is particularly evident is in the use of suprasegmental parts of speech, collectively known as prosody.

Prosody is commonly defined as encompassing suprasegmental features of speech such as intonation, stress, rhythm, and timing—elements that typically span across segments or syllables (Bussmann, 2006; Trask, 2004). These phenomena are often described as operating beyond the level of individual segments. However, prosody is not categorically separate from the segmental level. In fact, prosodic features are realized through phonetic parameters—including fundamental frequency (*f*0), duration, and intensity—which manifest physically in segmental material, particularly in vocalic nuclei.

Thus, while our analysis focuses on acoustic and articulatory characteristics within vowels (a segmental locus), the function and interpretation of *f*0 modulations and formant characteristics are inherently prosodic, especially when tied to intonational contour or expressive, iconic signaling. This reflects the well-established interaction between segmental and prosodic structure (e.g., Gussenhoven, 2004; Ladd, 2008), where prosodic patterns are phonetically realized within segments, and segments are shaped by their prosodic context. The flexibility inherent in this segmental-prosodic coupling—such as the ability to modulate *f*0 and formant frequencies—is made possible by human advanced voluntary control over vocal source and filter cues. These abilities appear to build on earlier non-verbal signaling systems for conveying body size and emotional states, while co-existing with a phylogenetically older repertoire of spontaneous calls (Anikin et al., 2023; Pisanski et al., 2016).

This paper delves into the iconic use of prosody, focusing on two notable cross-modal associations. The first examines the link between the vertical position of an object and *f*0, where high-pitched sounds are perceived as originating from higher vertical space, and speakers tend to use a higher fundamental frequency when discussing “higher” concepts (Clark et al., 2014; Mudd, 1963; Nygaard et al., 2009; Perlman, in press; Pratt, 1930; Roffler & Butler, 1968). The second explores the relationship between the size of an entity and vocal frequencies, where higher pitches are typically produced by and associated with smaller sources, and animals use fundamental frequency to signal size (Bee et al., 2000; Ohala, 1994). This association is not only about *f*0 but also involves formant frequencies, which can be indicative of size (Pisanski et al., 2014; Pisanski & Rendall, 2011).

The mechanisms underlying these cross-modal relationships are still debated. While some attribute them to biological constraints (larger animals have longer and heavier vocal folds and thus a lower *f*0; “frequency code” by Ohala, 1994), others point to influences from the natural environment (Parise et al., 2014), multisensory experiences related to synesthesia (e.g., Marks, 1975; Ramachandran & Hubbard, 2001), or semantic and affective reasons (for an overview, see McEwan et al., 2024). Building on the concept that sensorimotor processes might play a central role (Perniss & Vigliocco, 2014), our study investigates whether these cross-modal associations in prosody can be attributed to physiological sensorimotor dynamics. In contrast to many previous studies, we focus on the production of vocal frequencies (including *f*0 and formant frequencies) and their relation to the vertical position of an object and its size. Following classical semiotic theory (Peirce, 1955), we use *iconic* for cases where an acoustic dimension resembles an external property of the referent (e.g., higher *f*0 depicts vertical height), and *indexical* for cues that point to speaker-internal traits such as speaker size, vocal-tract length, or gender. In this view, the same acoustic parameter may carry both functions, but via distinct motor or articulatory routes.

By empirically testing these two relationships, we aim to shed light on the underpinnings of iconicity in prosody. This approach challenges the assumption that iconicity is merely an inherent and self-explanatory feature of language. Instead, it seeks to unravel the physiological and sensorimotor processes that may drive these iconic representations, contributing to a deeper understanding of the intersection between language, perception, and physical embodiment.

## MOTIVATION FOR THE CURRENT STUDY

### Iconicity of Vocal Frequencies

#### Vertical Position.

Envision a scenario where you hear someone shouting your name while walking down the street. Your instinctive response might involve turning towards the sound source, but can you discern from which floor the person is shouting? This query delves into the prosodic cues that underlie our perception of sound in the vertical dimension, a topic intriguing researchers for over a century (Seashore, 1899).

Pratt’s (1930) seminal work investigated the ability of listeners to localize sounds of varying fundamental frequencies in the vertical dimension. His findings, known as the Pratt effect, illustrate a tendency to associate higher pitches with elevated positions and lower pitches with lower positions. This phenomenon, originally demonstrated with octaves, has since been replicated with subtler frequency variations and across diverse populations, including congenitally blind individuals and children (Mudd, 1963; Roffler & Butler, 1968; Trimble, 1934).

Recent research by Parise et al. (2014) further explores this concept, examining how sound frequencies map to elevations in natural settings. Their findings corroborate the frequency-elevation mapping (FEM) effect, observing a strong correlation between high-frequency sounds and elevated sources, particularly in the 1–6 kHz range. This study highlights the role of the outer ear in enhancing high-frequency sounds and suggests that our spatial hearing might be attuned to the auditory statistics of our environment, shaping our expectations for sound localization. However, the applicability of the FEM effect to human speech perception remains an open question. Speech predominantly contains energy in lower frequency ranges (0–10 kHz, and often up to 1 kHz) (Johnson, 2011), and vertical sound localization relies on direction-dependent filtering of high-frequency components by the auricle, which may be less informative for speech than for other environmental sounds (Kandel et al., 2021; Köppl, 2009).

Studies investigating this phenomenon between vertical space and *f*0 in speech production have noted subtle *f*0 changes when conveying congruent prosodic information. For example, speakers tend to associate higher *f*0 for upward movements and lower *f*0 for downward movements in their speech (Ekström et al., 2022; Shintel et al., 2006). Such findings even extend to metaphors. Clark et al. (2014) observed a similar trend, with participants using higher *f*0 for stories with positive or upward metaphors and lower *f*0 for those with negative or downward metaphors. The mean *f*0 difference in these contexts was approximately 5.4 Hz, indicating that speakers integrate prosodic information congruent with their speech’s semantic content regarding spatial dimensions.

Nygaard et al. (2009) extended this understanding by investigating the role of *f*0 in conveying the meaning of novel words representing various adjective pairs, including spatial dimensions such as tall/short. Among their findings, participants used systematically higher *f*0 for words intended to mean “tall” compared to “short,” providing early evidence for iconic *f*0 use in vertical spatial dimensions, with listeners successfully interpreting these cues. The production of novel words resulted in mean *f*0 differences ranging from 25 Hz to 142 Hz, which were probably amplified by instructing participants to use infant-directed speech.

Two recent studies extended the exploration of prosodic iconicity to motion events. Ekström et al. (2022) tested if increasing and declining *f*0 can be iconically mapped to upward and downward movements. Their findings, while showing a limited effect, support the notion that prosody is iconically associated with motion, particularly with declining *f*0. This research enriches our perspective on how vertical space and prosodic elements like *f*0 interact, suggesting a broader iconic mapping beyond just spatial concepts.

Another line of research by Vainio and Vainio (2021) presented evidence that segmental elements of speech, such as vowels are linked to body actions between hand and mouth, proposing that sound symbolism, including prosody, may originate from embodied mechanisms. This suggests that the iconic prosody could be grounded in sensorimotor processes, resonating with our exploration of how vertical positioning influences and is influenced by prosodic features.

These findings, along with the contributions of recent studies, lead to several key insights. First, there is a perceptual association between high-frequency sounds and elevated positions (based on natural acoustic statistics), reflected in speech production when referencing elevated or lowered objects. The magnitude of this effect varies, with larger frequency differences typically observed in perception than in production (Clark et al., 2014; contrast with Nygaard et al., 2009). Second, the natural auditory landscape, where high-frequency sounds often originate from elevated sources, likely grounds this cognitive association (Parise et al., 2014). Moreover, studies such as those by Ekström et al. (2022) and Vainio and Vainio (2021) expand our understanding of prosodic iconicity, indicating that the mapping of prosodic elements to spatial and motion concepts may be influenced by sensorimotor processes. Our study aims to further elucidate these relations by exploring the physiological and sensorimotor processes that underlie iconic prosody.

#### Size.

Imagine encountering a stray dog in the park. Your emotional response might differ significantly based on whether the dog is large or small—but equally important is how the dog sounds. A large dog’s deep bark signals its size and potential threat level even before you see it, while a small dog’s high-pitched yapping immediately identifies it as less threatening. This instinctual reaction is rooted in a fundamental correlation between body size and vocal characteristics, and the relationship is so pervasive across species that Ohala (1984, 1994) formalized it as the “frequency code,” where lower frequencies signal dominance and larger size, while higher frequencies indicate submission and smaller size.

When perceiving animate beings, humans rely on vocal cues to estimate size. Formant frequencies significantly impact body size estimation, with lower formant dispersion consistently associated with larger speakers (Fitch, 1994). Notably, formant cues often supersede fundamental frequency cues in human body size perception (Pisanski & Rendall, 2011). Recent work by Anikin et al. (2022) demonstrates that listeners readily infer exaggerated body size or heightened aggression when the apparent vocal tract length is altered through static or dynamic formant scaling, pointing to the communicative power of voluntary vocal tract manipulation. However, our perceptual biases exceed actual biological correlations—a meta-analysis revealed only weak correlations between actual body size and formant frequencies, with marginal effects for fundamental frequency (Pisanski et al., 2014). This suggests our size perception system may be calibrated for communicative efficiency rather than strict accuracy.

The perceptual patterns of size estimation through the voice have biological foundations in animate beings. Larger organisms typically produce lower sounds due to longer, heavier vocal folds vibrating at slower rates (Gick et al., 2013; Riede & Brown, 2013). Adult male humans typically possess a considerably larger larynx than females, resulting in longer, more massive vocal folds that produce generally lower frequencies (Titze, 1989). This sexual dimorphism becomes especially pronounced during puberty (Fitch & Giedd, 1999). The vocal tract length similarly correlates with body size, affecting formant dispersion in predictable ways across species, including humans and rhesus macaques (Fitch, 1997).

Despite these biological constraints, vocal production in animals and humans demonstrates flexibility. Male frogs strategically lower their *f*0 when defending territories (Bee et al., 2000), showing that even non-human animals manipulate vocal parameters for communication, whether it is voluntary or not. Humans exhibit even greater control, modulating both static vocal indices that convey age and sex (Pisanski et al., 2014; Stathopoulos et al., 2011) and dynamic aspects expressing social and emotional states (Leongómez et al., 2021; Ponsot et al., 2018). Voice quality further extends this communicative capacity: Morton (1977) observed that in animal vocalizations, aggressive sounds typically have a low-frequency and harsh quality. These harsh qualities are produced by non-linear phenomena, i.e., irregular states in the spectrum such as frequency jumps, subharmonics, chaos, and biphonation that break the smooth harmonic structure of the voice (Anikin & Herbst, 2025), which can be interpreted as a signal of aggression and dominance in human vocalizations (Anikin et al., 2020, 2021). Conversely, higher, purer frequencies often appear in nurturing contexts like infant-directed speech (Fernald & Kuhl, 1987; Trainor & Desjardins, 2002).

The size-frequency relationship extends beyond animate beings to the perception of inanimate objects. Sapir’s (1929) seminal study revealed that listeners associate small entities with high-front vowels (/i/) and large entities with low-back vowels (/a/). This sound-symbolic relationship appears consistently across cultures, suggesting deep cognitive associations between acoustic properties and size perception regardless of the sound source. These associations may partially derive from the intrinsic pitch differences between vowels, where high-front vowels typically have higher fundamental frequency than low-back vowels (Chen et al., 2021; Whalen & Levitt, 1995).

Interestingly, inanimate objects also influence vocal production. Recent research by Vainio and colleagues further explores the relationship between size and vocal characteristics in inanimate objects. Vainio (2021) found that spatial and temporal magnitudes affect vowel production, with short distances and durations aligning with vowels like [i] and [e], and longer ones with [u], [æ], and [y]. Vainio, Mustonen, et al. (2019) demonstrated that number magnitude influences vocal responses, with larger numbers eliciting longer, higher-pitched vocalizations. Their other studies (Vainio et al., 2017; Vainio, Vainio, et al., 2019) revealed that the grasp-related size of objects and perceived grasp shape also influence vowel pronunciation, associating smaller objects and precision grip stimuli with vowels like [i], and larger objects and power grip stimuli with vowels such as [α] and [o]. These bidirectional effects, where perception influences production and vice versa, are further demonstrated by Parise and Spence (2012), who showed how visual size perceptions interact with auditory perceptions to shape vowel articulation.

In summary, the relationship between size and vocal characteristics operates through multiple mechanisms. At the biological level, larger organisms naturally produce lower-frequency sounds due to longer vocal folds and resonating cavities (Gick et al., 2013; Riede & Brown, 2013), creating reliable acoustic cues for size estimation. This relationship has become so deeply ingrained in perception that humans consistently associate lower frequencies with larger entities and higher frequencies with smaller ones, even when judging inanimate objects (Sapir, 1929). Most significantly for our hypotheses, this relationship manifests bidirectionally—not only do we perceive size from vocal properties, but our vocal production is influenced by perceived size, with speakers modifying both fundamental frequency and articulation when referencing differently sized entities (Vainio et al., 2017; Vainio, Vainio, et al., 2019). These findings provide a foundation for investigating how object size might influence specific acoustic parameters in speech production.

### The Influence of Physiology on Vocal Behavior

The evidence so far demonstrates how vocal behavior iconically reflects real-world aspects such as vertical position or entity size. Delving deeper, we focus on the physiological effects shaping vocal behavior, particularly *f*0 patterns influenced by speech organ morphology.

*f*0 control in speech, an anatomically complex process, is linked with respiration, which serves both life-sustaining and speech functions (Anokhin, 1974; Perkins & Kent, 1986). Vocal-fold oscillations can be explained by the myoelastic–aerodynamic theory (van den Berg, 1958) whereby subglottal (lung) pressure builds beneath the adducted folds, forcing them apart; once air rushes through the glottis, the resulting drop in pressure (Bernoulli effect) and the elastic recoil of the tissue pulls the folds back together. This cycle of opening and closing, aided by the mucosal wave travelling over the fold surface, sustains a periodic vibration. The vibration frequency is determined by the pressure difference and by both intrinsic and extrinsic laryngeal muscles (Erickson et al., 1982).

The vocal frequency is largely governed by the activity of laryngeal muscles. The cricothyroid muscle (CT), a key intralaryngeal muscle, is central to controlling the *f*0. Activation of the CT muscle results in tensioning of the vocal folds, leading to higher-frequency sounds (Gick et al., 2013). Additionally, the thyroarytenoid (TA), lateral cricoarytenoid (LCA), and the cricopharyngeus (CP) muscle influence *f*0 (see Honda, 1988 for details). Furthermore, strap muscles contribute to lowering *f*0 in conjunction with the CT muscle (Erickson et al., 1982; Honda, 1996; Hong et al., 1997). The impact of external movements, like head rotation, on the larynx position and subsequently on *f*0 control, underscores the complexity of the vocal system.

When a wolf howls, it raises its head upwards. It is, on the one hand, to make the sound spread further, but on the other hand, the movement may allow the wolf to achieve a higher vocal frequency. Similarly in humans, the head position seems to be used by popular artists to reach especially high notes and hold them^1^, which is also helpful in perception as shown by Chen and Massaro (2008) in a study where the visible information of neck and head movements improved the ability of Mandarin speakers to recognize the lexical tone.

A study by Munhall et al. (2004) found that speaker head movement during speech production can explain 63% of the variation in the *f*0 using a sentence-by-sentence multiple regression analysis. The study demonstrated a direct correlation between head movements and *f*0 changes, with elevation correlating with *f*0 increase and lowering with *f*0 decrease, indicating that visual prosody significantly contributes to speech in a manner akin to acoustic prosody. In a recent study, Carignan et al. (2024) further substantiated that downward head nods and *f*0 are coordinated in the production of prosodic prominence in French speakers. They also reported individual strategies, but as a general finding they found that co-speech head nods magnify prosodic prominence in the production of contrastive and narrow focus in French.

Adding to this understanding, Liu et al. (2020) directly explored the relationship between vertical head movement and *f*0 in speech, comparing congenitally blind and sighted speakers. The research found positive correlations between *f*0 and vertical head movement for both groups, with a stronger correlation in blind speakers. Interestingly, sighted speakers displayed larger head movements and a higher head movement per semitone ratio. These results suggest that physiological processes contribute to *f*0-related head movement and that sighted speakers use visual cues to communicate *f*0 information through enhanced head movement. The study underscores the multimodal nature of speech and the potential physiological basis of *f*0 control.

Fundamental frequency is pivotal in vocal expression, yet formant frequencies and their underlying production are equally vital, particularly concerning the perceived size of entities. Vocal iconicity studies, such as those by Sapir (1929), highlight a preference for associating larger objects with open vowels and smaller ones with closed vowels, influenced by lip opening and formant variations. Thus, one physiological aspect of vocal iconicity potentially related to size is lip opening, with larger objects associated with greater lip openings and smaller objects with lesser openings. However, most demonstrations of size-related sound symbolism compare acoustically distant vowel categories (e.g., /i/ vs. /a/; Sapir, 1929); evidence for systematic modulation within a single vowel category is unknown. Within a single vowel sound, formants show tolerance due to coarticulation, offering variability (e.g., Öhman, 1966; Strange & Bohn, 1998). This variability in formants can serve as iconic signals for the size of the object referred to. Formant dispersion, measured as the averaged difference between successive formant frequencies (Fitch, 1997), is another key acoustic feature, and has been linked to perceived size. Because formant dispersion reflects vocal-tract length, it can, in principle, cue perceived size; whether such cues vary within a vowel remains an open question. Combining lip opening and formant dispersion provides a comprehensive view of vocal production, capturing both visible and invisible articulatory dimensions.

## HYPOTHESES

Drawing on the literature on iconic prosody and speech organ morphology, we developed two hypotheses to explore the physiological basis for iconicity.

Hypothesis 1 (H1) examines the influence of head position on *f*0, positing that upward head rotation elevates *f*0. This hypothesis aims to clarify the link between an object’s vertical position and *f*0, testing if an upward-looking head position induces an iconic cross-modal effect, with an expected *f*0 increase due to the engagement of larynx-related muscles (Erickson et al., 1982; Honda, 1996; Liu et al., 2020).

Hypothesis 2 (H2) is divided into two parts to comprehensively explore the relationship between object size, lip opening, and formant frequencies. Hypothesis 2a (H2a) posits that the size of the object being named will influence the degree of lip opening, with larger objects leading to greater lip opening. This part of the hypothesis builds upon existing research linking lip opening to object size perception (shown across vowels differing in lip opening by Sapir, 1929; Vainio, 2021). Hypothesis 2b (H2b) focuses on formant dispersion, proposing that larger objects will result in decreased formant dispersion, in contrast to smaller objects. This part of the hypothesis is grounded in research showing that formant dispersion reflects vocal tract length, with larger formant dispersion indicating shorter vocal tracts and thus smaller perceived size, while smaller formant dispersion indicates longer vocal tracts and larger perceived size (Fitch, 1997). By examining these aspects, H2 seeks to provide a deeper understanding of how object size impacts specific articulatory parameters in speech production, contributing to the field of vocal iconicity.

## METHODOLOGY

### Participants

In this study, we exclusively recruited female participants to eliminate gender differences in fundamental frequency as a potential confounding factor. This decision was particularly relevant to our initial plan of analyzing the data with linear mixed-effects models and minimizing the number of predictors and their potential interactions. Initially, 31 native German speakers participated, but due to a technical issue with the recording equipment for one participant, we included data from only 30 in our analysis. The participants had a mean age of 27.77 years (ranging from 19 to 48 years) and an average height of 167.3 cm (spanning from 152 cm to 183 cm). The majority were right-handed (29 out of 30 participants). Recruitment was facilitated through the Leibniz-Centre General Linguistics’ participant database. Each participant provided written consent after receiving basic information about the task and was compensated for their contribution. This project received approval from the Ethics Commission of the German Society of Linguistics. The data and scripts from this study are openly accessible in the OSF repository: https://osf.io/e3cb4/

### Experimental Procedure

**Figure 1.**
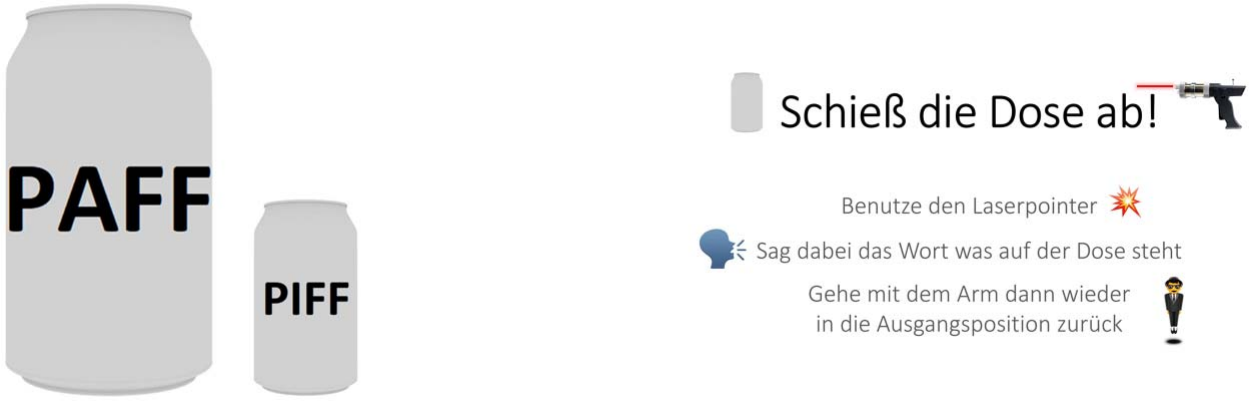
(left) Large and small cans in comparison. A large can was proportionally twice the size of a small can, as measured by the height of the can. The cans displayed either piff [pɪf] or paff [paf]. (right) An instruction slide shown to the participants. The participants were asked to move their arm back to the onset position after each “shot”.

A novel experimental paradigm was devised to investigate the impact of vertical head position and lip opening on prosodic parameters and their iconicity. The task involved a game-like scenario, in which participants had to shoot cans projected onto a wall by pointing a laser pointer at the can and saying the word written on it. To avoid learning effects, two different onomatopoeic words, *piff* [pɪf] or *paff* [paf], were randomly assigned to appear on the cans. The cans came in two sizes, small and large, with the larger can being twice the size of the small can (Figure 1A). The cans were arranged in five equidistant vertical and horizontal levels and projected onto a 1.30 × 1.30 m area, with the participant positioned 1 m away. The vertical position of the cans was designed to elicit different vertical head positions, ensuring head movement was involved in the task. Each participant completed 100 trials, with the task lasting approximately 5–6 minutes.

After the participant pointed the laser pointer at the can and said the assigned onomatopoeic word, the can was animated to simulate being “shot” and subsequently dropped downward, mimicking the effect of being hit. A blank white screen then appeared briefly before the next can was presented. The presentation order was pre-randomized to introduce unpredictability and avoid order effects, with five datasets created for this purpose. The main task was preceded by a short familiarization phase consisting of five cans bearing words other than *piff* or *paff*. The instructions provided to the participants are presented in Figure 1B, with the only specific instruction being to return the arm to the starting position after each trial. The experimental design is based on two iconic relationships: between vertical space and *f*0 (pitch), and between size and formant frequencies. The paradigm also incorporates a physiological component, with head position being a component of *f*0 and lip opening being a component of *f*1. The use of a large surface for projecting the targets was intended to evoke head position change, with the participant unable to solely rely on eye movements to target the cans. The inclusion of two visibly different can sizes was intended to elicit different lip openings.

### Data Recording

**Figure 2.**
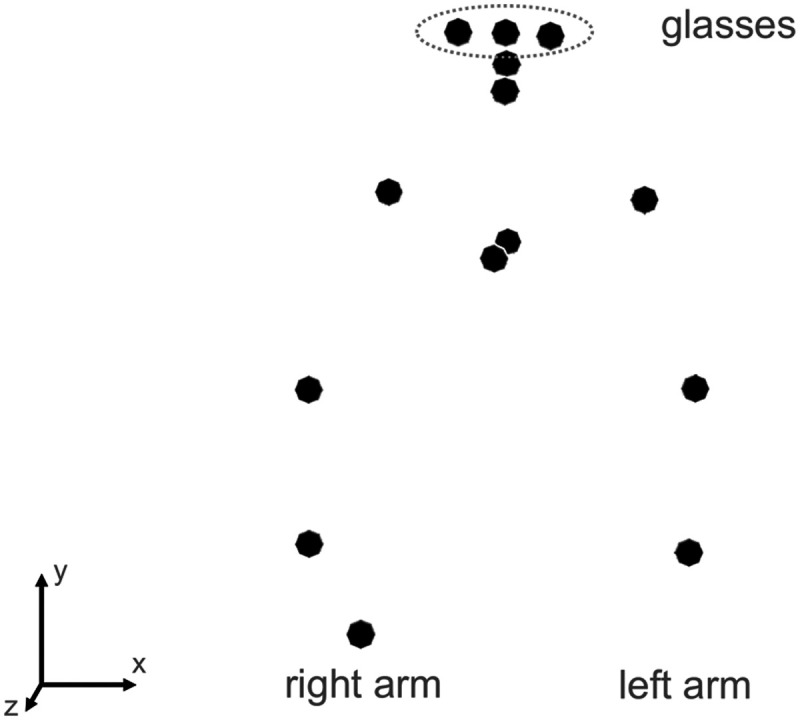
The placement of the motion capture markers. Here, it can be seen that the participant is holding the laser pointer in her right hand.

During the experiment, participants’ voice was recorded using a Sennheiser ME 64 cardioid microphone. Acoustic data was recorded with a sampling rate of 44.1 kHz with 16-bit resolution and a bit rate of 705 kBit/s. Additionally, the participants’ movements were tracked using an Optitrack motion capture system, which consisted of 12 Prime 13 cameras and Motive software version 1.9.0. The system’s precision was 0.3 mm after calibration, and motion was captured at a sampling frequency of 120 Hz. While tracking the motion of the head and lip opening was the primary focus to test H1 and H2, 14 markers were placed on various upper body parts as reference points and to allow for additional measurements, such as head pitch angle and arm movement. These markers included three on a pair of lensless glasses (left corner, center, and right corner), one on the upper and lower lips, one at the sternum position, one at the location of the fourth thoracic spine vertebra, one on the laser pointer, and symmetrically two on the shoulders, elbows, and wrists. The placement of all markers is illustrated in Figure 2.

### Data Processing and Analysis

#### Data Processing.

The acoustic data were labeled automatically at the phoneme level, and subsequently corrected manually using Praat (Boersma & Weenik, 2018), after being processed by WebMAUS (Kisler et al., 2017). Parameters for fundamental frequency (*f*0) and the first three formants (*f*1–*f*3) were automatically extracted using a Praat script. The onset and offset of the vowel were defined as the onset and offset of vocal fold oscillations, respectively. The mean fundamental frequency in hertz was calculated for the entire vowel interval, and median values in hertz for formants, also for the whole vowel interval. To avoid pitch tracker octave jumps, commonly seen with creaky voice, the fundamental frequency range during automatic extraction was restricted to 150–400 Hz. For each token, we also computed formant dispersion as

$$f_{\text{disp}}=\frac{\left(F_{2}-F_{1}\right)+\left(F_{3}-F_{2}\right)}{2}$$

providing a single measure of average spacing between the first three formants.

The position of the markers was measured in a three-dimensional space, using ground level as a reference for the *y*-axis. We processed the motion capture data by first manually renaming each motion capture marker for consistency across participants using Mokka (version 0.6.2; Barre & Armand, 2014) and saving the data as *.csv* files. We further processed the data using MATLAB (version R2017b) for the distance between the lips and in R (version 4.4.1) for the vertical position of the head. The degree of lip opening was calculated frame by frame as the Euclidean distance between the lower and upper lip sensors in the 3D space (with x_1_, y_1_, z_1_ corresponding to the upper lip marker and x_2_, y_2_, z_2_ to the lower lip marker):

$$lip\;\text{opening distance}=\sqrt{\left({\left(x_{1}+x_{2}\right)}^{2}+{\left(y_{1}+y_{2}\right)}^{2}+{\left(z_{1}+z_{2}\right)}^{2}\right)}$$

We then searched automatically for the maximal lip opening distance within the respective acoustically defined vowel interval.

Additionally, we determined the maximal vertical position of the wrist of the pointing hand during the word interval to serve as a measure of arm elevation, which could potentially influence our dependent variables. The marker on the laser pointer was deemed unreliable for this purpose, as it was occasionally hidden for some participants.

To parameterize head position, we obtained three measures: The maximal vertical position of the center of the glasses marker, the relative vertical position of the center of the glasses marker (head) to the marker at the fourth thoracic vertebra, and the head pitch angle. In motor control, the rotation of the head in this direction is called pitch. However, we will hereafter call it head rotation angle to avoid confusion with pitch being associated with the acoustic measure fundamental frequency (perceptually pitch). The head rotation angle was the angle between two vectors: a reference vector along the body (from sternum → to the fourth thoracic vertebra), and a target vector representing head orientation (from the fourth thoracic vertebra → to the mid glasses marker). The head rotation angle captures how much the head tilts (nods) relative to the trunk. It was computed via the dot product of the reference vector (v_1_) and the target vector (v_2_):

$$\theta =\text{arccos}\left[\left(v_{1}\cdot v_{2}\right),/,\left(\left\|v_{1}\|\|v_{2}\right\|\right)\right]$$

**Figure 3.**
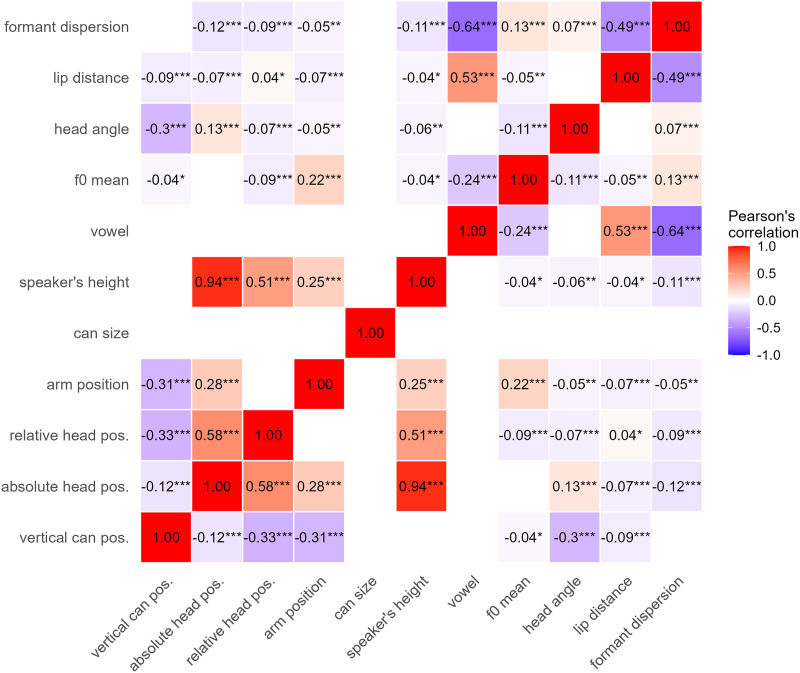
Correlation matrix showing significant pairwise relationships between behavioral and acoustic variables. Non-significant correlations are omitted for clarity. Significance levels: *p < 0.05, **p < 0.01, ***p < 0.001. Color represents correlation direction and strength.

All head position variables were calculated for the acoustically defined interval of the whole word (either *piff* or *paff*), and we took the maximum value for each variable in this interval. The head rotation angle is the most biomechanically inspired measure (Winter, 2009), taking head rotation towards the can into account. Vertical head position may be the least reliable measure because it is an absolute measure that can also be affected by any motion of the trunk that carries the head. For the relative vertical head position, we took a relatively stable reference position into account and calculated the vertical distance between the head and the back; it may therefore be a more reliable measure. Using correlation analysis, these multiple head position variables were tested for potential multicollinearity concerns: absolute head position correlated with speaker height (*r* = 0.94, *p* < 0.001) and with relative head position (*r* = 0.58, *p* < 0.001), relative head position showed moderate correlations with vertical can position (*r* = –0.33, *p* < 0.001) and speaker’s height (*r* = 0.51, *p* < 0.001), and head rotation angle showed a moderate correlation with vertical can position (*r* = –0.30, *p* < 0.001). Figure 3 shows a correlation matrix with significant correlations among the variables. To select the optimal head position metric, we compared three models, each including one of the head position metrics plus vertical position of the can as predictors, using leave-one-out cross-validation (LOO-CV) with Pareto smoothed importance sampling (Vehtari et al., 2017). Model comparison favored head rotation angle as the most predictive metric, while avoiding the severe collinearity issues that would arise from including speaker’s height-correlated measures, such as absolute or relative head position. Head rotation angle also provided the most precise physiological interpretation, representing the rotational component of head position most directly linked to laryngeal muscle engagement.

As mentioned in Section II A, we excluded data from one participant due to a technical error. Therefore, there were a total of 3,000 data points (100 per speaker). Measurement errors were identified and removed by grouping the data by participant and vowel, and then excluding measurements beyond two interquartile ranges (IQRs). The amount of missing data increased slightly after this procedure, in the acoustic parameters: in *f*0 from 2.16% to 4.3%, in *f*1 from 0.36% to 1.43%, in *f*2 from 0.03% to 1.87%, and in *f*3 from 0.03% to 1.5%; in kinematic parameters: in angle from 0% to 0.13% and no change in lip opening (NA = 1%).

#### Statistical Modeling.

Statistical analyses were conducted in R (version 4.4.1; R Core Team, 2024). Data processing was conducted with the *tidyverse* package (version 2.0.0; Wickham, 2017), and data visualization was performed using *ggplot2* (version 3.5.1; Wickham, 2016). Bayesian hierarchical modeling was conducted using the *brms* package (version 2.22.0; Bürkner, 2017).

Model comparison was performed using leave-one-out cross-validation (LOO-CV), which estimates out-of-sample predictive performance without refitting models. LOO-CV iteratively “leaves out” each observation and assesses the model’s ability to predict that observation using the remaining data (Vehtari et al., 2017). Rather than literally refitting models N times, we used Pareto smoothed importance sampling (PSIS-LOO), which reweights existing posterior samples to approximate what would happen if each observation were removed, providing an efficient approximation while offering diagnostic information about model reliability.

During initial data exploration, we observed individual differences in head movement patterns across speakers, with some exhibiting larger movement ranges than others. To test whether these individual differences moderated the head rotation angle effects, we classified speakers into “movers” and “non-movers” based on the standard deviation of their head rotation angle movements across trials. Using the mean standard deviation (2.405°) as a cutoff, speakers with higher variability (n = 11) were classified as “movers” and those with lower variability (n = 19) as “non-movers.” This classification allowed us to test whether the head angle effect on *f*0 differed between speakers who generally move their heads more versus less during speech production.

Categorical predictors (vowel, can size, and mover type) were sum-coded, where each level received values of +0.5 and −0.5, ensuring that model intercepts represented grand means and avoiding the arbitrary reference category inherent in treatment coding. Continuous predictors (vertical position, head angle, arm position) were mean-centered.

For H1 (*f*0 mean), we used a lognormal family. For H2 (lip opening and formant dispersion), we determined the optimal likelihood family by comparing intercept-only models across different distributions using LOO-CV. We tested gaussian, gamma (log link), and lognormal families for both H2 outcome variables. H2a (lip opening) was best fit by the lognormal family, while H2b (formant dispersion) was optimally modeled using the Gaussian family.

We used weakly informative priors that provide gentle regularization without overwhelming the data. For lognormal models, intercept priors were centered on the log-transformed mean of the outcome variable with moderate uncertainty (*SD* = 0.5 for H1, *SD* = 1.0 for H2a), slope priors were centered at zero with modest spread (*SD* = 0.5 for H1, *SD* = 1.0 for H2a), and residual standard deviations received exponential(1) priors. For the Gaussian model (H2b), intercept priors were centered on the untransformed outcome mean with *SD* = 1.0, slopes were normal(0, 10), and residual standard deviations were exponential(1). These priors are weakly informative because they gently constrain parameters to reasonable ranges while allowing the data to dominate the posterior inference.

#### Model Comparison Procedure.

To systematically evaluate competing predictors and to address the potential confounding between head position and vertical can position, we employed a hierarchical model comparison approach using leave-one-out cross-validation (LOO-CV) with Pareto smoothed importance sampling (Vehtari et al., 2017). For both H1 (outcome variable: *f*0 mean) and H2 (outcome variables: lip opening and formant dispersion), we fitted a series of nested models with increasing complexity and selected the model with the highest expected log predictive density (ELPD), representing the optimal balance between model fit and complexity.

For H1, the hierarchical comparison proceeded in three blocks: Block A tested individual predictors (vertical position of the can, head rotation angle, arm position) and an head angle × mover interaction alongside vowel as a control variable. Block B included both vertical position of the can and head rotation angle, compared to head rotation angle × mover interaction, alongside vowel as control. Block C additionally incorporated arm position. For H2, the comparison proceeded as follows: Block A.1 tested can size and vowel main effects, Block A.2 added a can size × vowel interaction, and Block B incorporated angle as a control variable alongside the main effects.

Models within each block were compared using ELPD differences, and we report ELPD differences and their standard errors below without applying arbitrary cutoff thresholds, following best practices outlined by (Sivula et al., 2025). All models used moment-matched importance sampling to ensure stable LOO estimates for observations with high Pareto k values (k > 0.7). For H1, we compared eight candidate models, plus one intercept-only model, while for H2, we compared three candidate models, plus one intercept-only model, for each outcome variable.

Crucially, this approach allowed us to directly compare the predictive performance of head rotation angle versus vertical can position in H1, addressing our concerns about the correlation between these variables (*r* = −0.13, *p* < .001). Rather than fitting separate models for the correlated predictors, the hierarchical comparison provides evidence for which predictor better captures the underlying mechanism predicting acoustic and articulatory variation through comparison of different modeling scenarios.

## RESULTS

### H1: Head Position and *f*0

**Figure 4.**
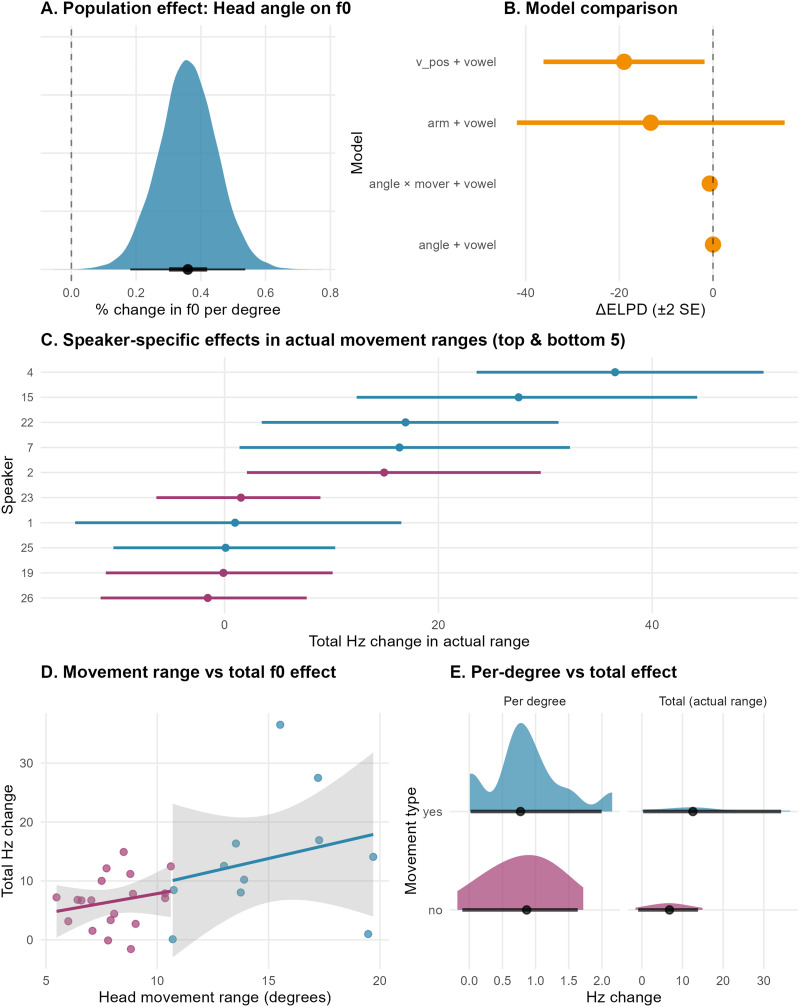
(A) Posterior distribution of the head rotation angle effect showing a reliable 0.36% increase per degree (95% credible interval entirely above zero). (B) Model comparison demonstrating superiority of head rotation angle over can vertical position (Δ ELPD = 19) and arm position (Δ ELPD = 13). (C) Individual speaker total *f*0 effects across actual movement ranges, with movers (blue) and non-movers (pink) showing substantial individual variation (range: −1.6 to 36.5 Hz). (D) Positive relationship between head movement range and total *f*0 effect, with “movers” (blue) and “non-movers” (pink) following the same slope but operating in different ranges. (E) Per-degree sensitivity comparison showing nearly identical distributions between movers and non-movers (left), while total effects differ substantially due to movement range differences (right). Results demonstrate a consistent physiological mechanism across individuals.

Nine candidate mixed-effects models were compared using PSIS-LOO cross-validation to test H1. The model containing head rotation angle and vowel had the highest expected out-of-sample performance. In this optimal model, vowel showed the strongest effect on mean *f*0 (*β* = −19.0[−24.4, −13.6] Hz) with a posterior probability of 100% of a negative effect. On average, /a/ was 19 Hz lower than /ɪ/. Head rotation angle showed a reliable positive effect (*β* = 0.85[0.43, 1.27] Hz per degree) with a posterior probability of 100%. The head angle effect translates to an average 0.85 Hz per degree of upward rotation, representing a 0.36% increase in *f*0 mean per degree (cf. Figure 4A). While vertical can position also showed a reliable positive effect when included as the sole predictor (posterior probability = 100%), producing 1.22 Hz [95% CrI: 0.60, 1.86] increase per 32.5 cm step (0.038 Hz per cm), the vertical can position-only model was decisively worse at explaining the data overall (Δ ELPD = −19.0 ± 8.6; cf. Figure 4B for model comparison). The geometric relationship between these measures explains why both show reliable effects: with participants ~1 m from the wall, a 10° head rotation corresponds to ~17.6 cm vertical displacement (tan(10°) × 100 cm); head rotation angle predicts 8.5 Hz change while vertical can position predicts only 0.67 Hz for this same physical movement and we find the evidence for the former in our data. This demonstrates that head rotation angle, as the direct physiological measure, captures more variance than its geometric projection and represents the primary mechanism predicting *f*0 variation.

The head rotation angle effect is statistically robust and represents a small to medium effect size. In musical terms, the effect corresponds to approximately 6 cents per degree at the baseline *f*0 of 236 Hz, and across a head movement of 10 degrees would produce about 8.5 Hz change. Relating this effect to real in-experiment behavior, individual speakers showed head rotation movement ranging from 5.47 to 19.7 degrees, with corresponding total mean *f*0 effects ranging from −1.57 to 36.5 Hz (cf. Figure 4C). The median individual head rotation range was approximately 8–9 degrees, producing typical total effects of 3–15 Hz. Across the complete observed range of head rotations (33.1°), the population effect corresponds to 29.8 Hz [14.7, 45.9 Hz], representing a 12.6% [6.2%, 19.4%] change. The model demonstrated very good predictive accuracy with a Bayesian R^2^ of 0.802, indicating that head rotation angle and vowel together explain approximately 80% of the variance in fundamental frequency. Model diagnostics confirmed adequate convergence (all Rhat = 1.00) with sufficient effective sample sizes.

Adding an angle × mover interaction reduced ELPD by only 0.7 (*SE* 0.2), indicating no meaningful moderation by individual movement propensity. The posterior median difference between mover and non-mover per-degree sensitivities was minimal at 0.028% [−0.139%, 0.198%] per degree with a posterior probability of a positive effect of 63% that movers show larger per-degree effects. However, movers exhibited greater movement ranges: non-movers averaged 8.0 degrees range (equal to an average of 6.5 Hz total effect) while movers averaged 15.0 degrees (equal to an average of 13.8 Hz total effect). Figures 4D and 4E demonstrate that the slope of the effect is the same between movers and non-movers, but the movement range modulates the magnitude of the effect.

When the model included vertical arm position (marker on the hand pointing towards the can) as a predictor of *f*0 mean instead of head angle, it visibly reduced the performance (Δ ELPD = −13.3 ± 14.3), suggesting that vertical arm position does not contribute meaningfully to explain mean *f*0 variation beyond potential artifacts of correlated movement. Models incorporating additional interactions or combinations of predictors were penalized by more than 200 ELPD points.

These results confirm that upward head rotation increases fundamental frequency, with head angle demonstrating substantially better predictive performance than vertical position of the can itself. The effect operates consistently across participants regardless of their general tendency to move their head, with individual differences in total effect magnitude driven by variation in movement amplitude.

### H2: Object Size and Lip Opening/Formant Frequencies

Eight candidate mixed-effects models were compared using PSIS-LOO cross-validation to test H2. For predicting lip opening, the model containing can size and vowel main effects had the highest expected out-of-sample performance. In this optimal model, vowel showed the strongest effect on lip opening (*β* = 0.45[0.39, 0.50] mm) with a posterior probability of 100% of a positive effect. On average, /a/ caused 0.45 mm larger lip opening than /ɪ/. Can size showed no reliable effect (*β* = 0.001[−0.011, 0.012] mm) with a posterior probability of 56% for a positive effect. For predicting formant dispersion, the model containing can size and vowel main effects similarly had the highest expected out-of-sample performance. Vowel showed a strong negative effect on formant dispersion (*β* = −55.5[−73.9, −36.3] Hz) with a posterior probability of 100%. On average, /a/ had 55.5 Hz lower formant dispersion than /ɪ/. Can size showed no reliable effect (*β* = 1.3[−2.7, 5.4] Hz) with a posterior probability of 74% for a positive effect.

The vowel effects represent large effect sizes for both outcomes. For lip opening, the vowel effect corresponds to a 10.5% increase from /ɪ/ to /a/ on average, reflecting the natural articulatory differences between these vowels. For formant dispersion, the vowel effect represents an average 5.3% decrease from /ɪ/ to /a/. In contrast, can size effects were negligible for both outcomes, representing minimal change and no reliability in the direction of the effect. The lip opening model demonstrated very good predictive accuracy with a Bayesian R^2^ of 0.90, while the formant dispersion model showed good predictive accuracy with a Bayesian R^2^ of 0.79. Model diagnostics confirmed adequate convergence with sufficient effective sample sizes for both outcomes.

Adding can size × vowel interactions reduced ELPD by only 0.9 (*SE* 0.4) for lip opening and 0.8 (*SE* 0.6) for formant dispersion, indicating no meaningful interaction effects for either outcome. The posterior evidence ratios for can size effects were weak (0.41 for lip opening, 0.45 for formant dispersion), providing inconclusive evidence for any object size influence on articulatory and acoustic parameters.

When models included head rotation angle as a control variable, performance was drastically reduced for lip opening (Δ ELPD = −198.4 ± 28.2) and formant dispersion (Δ ELPD = −747.6 ± 40.4), indicating these predictors add complexity without improving prediction. Similarly, including arm position as a control variable substantially worsened model performance, suggesting that postural variables do not contribute meaningfully to explaining articulatory variation beyond potential artifacts of correlated movement.

These results do not support H2, as object size showed no detectable effects on either lip opening or formant dispersion. Instead, vowel identity overwhelmingly dominated both articulatory and acoustic outcomes, with can size effects remaining negligible. The findings suggest that, contrary to our hypothesis, perceived object size does not influence lip opening or vocal tract configuration in this experimental paradigm.

## DISCUSSION

This study set out to investigate the physiological underpinnings of iconic prosody by examining two primary hypotheses. Hypothesis 1 proposed that upward head rotation towards an object placed in vertical space would lead to an increase in fundamental frequency (*f*0), thereby linking an object’s vertical position with *f*0 through physical head position change. Hypothesis 2 suggested that the size of an object influences articulatory parameters: larger objects would result in greater lip opening (H2a) and increased formant dispersion (H2b).

The findings offer substantial support for Hypothesis 1, demonstrating a robust and reliable relationship between head rotation angle and *f*0. In contrast, we found no support for Hypothesis 2, as object size showed no detectable effects on either lip opening or formant dispersion.

### Head Position and *f*0: Physiological Mechanisms of Iconic Prosody

The analysis of H1 revealed that head rotation angle is a reliable predictor of mean *f*0, with an average increase of 0.85 Hz for every degree of upward head rotation. This effect was robust across participants, with a posterior probability of 100%, indicating that the elevation in fundamental frequency is a consequence of the physical act of raising the head. Importantly, while vertical position also showed a reliable effect when included as the sole predictor (posterior probability = 100%), producing a 1.22 Hz increase per 32.5 cm step, our model comparison approach demonstrated that head angle substantially outperformed vertical position of the object itself in predicting mean *f*0 variation (ΔELPD = −19.0 ± 8.6). This difference can be understood through the geometric relationship between head angle and vertical position of the can: with participants positioned approximately 1 meter from the wall, a 10° head rotation corresponds to roughly 17.6 cm vertical displacement. Head angle predicts an 8.5 Hz change, while vertical position predicts only 0.67 Hz for this same physical movement. This demonstrates that head angle, as the direct physiological measure, captures substantially more variance than its geometric projection and represents the primary mechanism predicting mean *f*0 variation.

The physiological basis for this effect lies in the engagement of laryngeal muscles during upward head movement. When the head rotates upward, several anatomical changes occur that directly influence vocal fold tension and vibration frequency. The CT muscle, which is central to *f*0 control through its regulation of vocal fold tension, is likely engaged differentially during head rotation (Gick et al., 2013). Additionally, the strap muscles, which contribute to *f*0 modulation in conjunction with intrinsic laryngeal muscles (Erickson et al., 1982; Honda, 1996), may be stretched or activated during upward head movement, creating mechanical changes that systematically alter laryngeal configuration.

This result is consistent with studies by Munhall et al. (2004), who found that head movements correlate with changes in *f*0 during speech, Carignan et al. (2024), who reported coordination between *f*0 and head movement for enhancing prominence, and Liu et al. (2020), who demonstrated a positive correlation between vertical head movement and *f*0 in both congenitally blind and sighted speakers. These studies suggest that the act of raising the head engages laryngeal muscles, correlating with an increase in *f*0—a physiological basis for the iconic association between upward movement and higher *f*0.

The head-induced *f*0 rise, therefore, fits the iconic pattern: vertical head movement elevates the larynx, creating an *f*0 contour that resembles the spatial height of the target. This represents a perception-production link from spatial perception to vocal production, where the physical act of looking upward is connected with the acoustic pattern associated with height. While *f*0 can be consciously controlled, to some extent, this mechanism appears to operate below the threshold of conscious control, as evidenced by its consistency across participants regardless of their propensity to move their heads. Individual speakers showed head movement ranges from 5.47 to 19.7 degrees, with corresponding total mean *f*0 effects ranging from −1.57 to 36.5 Hz, yet the per-degree sensitivity remained remarkably consistent between movers and non-movers (posterior median difference of only 0.028% per degree).

This physiological coupling provides a mechanistic explanation for the widespread cross-linguistic tendency to associate higher pitch with spatial height (Parise et al., 2014; Pratt, 1930). Rather than being an arbitrary cultural convention, the height-pitch mapping appears to be grounded in the biomechanical constraints of the vocal system. When humans direct their attention upward, the resulting head position change is automatically associated with a higher pitch, creating a natural foundation for iconic sound symbolism that transcends individual languages and cultures.

The effect size, while modest in absolute terms, represents a meaningful acoustic change. In musical terms, the 0.85 Hz per degree effect corresponds to approximately 6 cents per degree at the baseline mean *f*0 of 236 Hz. Across a typical head movement range of 8–9 degrees, this corresponds to 3–15 Hz changes, representing perceptible pitch variations that could contribute to communicative effectiveness. The model predictive accuracy demonstrates that head angle and vowel together explain approximately 80% of the variance in mean *f*0 in our task, highlighting the primary role of these factors in *f*0 control.

### Object Size and Lip Opening/Formant Dispersion Parameters: The Limits of Iconic Influence

In examining Hypothesis 2, the results did not align with the initial predictions. Despite strong effects of vowel type on lip opening (H2a) and formant dispersion (H2b) – with /a/ yielding ~10.5% larger lip openings and ~5.3% lower formant dispersion compared to /ɪ/ – there was no reliable effect of object size on either of the outcome variables. Both lip opening and formant dispersion were purely indexical in our data, tracking vowel-specific articulatory configurations rather than the external size of the cans. The dominant role of vowel identity in determining articulatory configurations underscores a hierarchical nature of speech production, where phonemic requirements take clear precedence over potential iconic adjustments. The vowel effects represent large effect sizes that reflect fundamental articulatory requirements for vowel production (Fant, 1970; Mooshammer & Geng, 2008), with /a/ requiring greater lip opening and lower formant dispersion compared to /ɪ/ due to its more open vocal tract configuration.

Under the within-vowel conditions tested here, we found no detectable influence of object size on lip opening or formant dispersion. This result suggests that size-related sound symbolism may attenuate when articulatory variation is confined to individual vowel categories. While vowel /ɪ/ may have been further constrained by the dense high-front vowel system of German, which leaves little room for additional lip opening variation (without lip protrusion), this should be less the case for /a/, which is more free to vary. Vowel space density in German could therefore not be the only reason why no effect was found for single categories.

Another potential explanation might be animacy. Many studies referring to size effects are discussed in line with animate entities, while the cans that are depicted in our study are inanimate. It is well-established that formants can effectively signal size in human vocal communication. While González (2004) found only weak passive correlations between formant frequencies and body size in adult humans, Pisanski et al. (2014) demonstrated that formants serve as reliable cues to speaker size. However, this formant-based size signaling apparently does not extend to objects that speakers refer to. The vocal system appears capable of using formant dispersion to convey information about the speaker’s own characteristics, but this indexical signaling capacity does not readily transfer to iconic representation of external object properties.

### Implications for Iconicity Theories

Collectively, the results highlight the role of head position in modulating fundamental frequency, supporting the notion that upward head rotation can enhance *f*0 and contribute to iconic prosody. However, the expected effects of object size on articulatory parameters were not observed, indicating that the relationship between object size and speech production may be more complex than initially hypothesized. Our findings illustrate how a single utterance can multiplex *iconic* information about the referent (pitch-height mapping) with *indexical* information about the sound (lip opening and formant dispersion), showing the multifunctional nature of prosody. The relationship between head position and *f*0 reinforces the idea that sensorimotor processes contribute to the iconic mapping between physical space and *f*0, supporting theories that posit a connection between bodily properties and linguistic expression (Ohala, 1994; Perniss & Vigliocco, 2014). This suggests that iconicity arises not just from abstract associations but from concrete physiological mechanisms.

Our findings reveal constraints on vocal iconicity within linguistic systems. The robust head angle-*f*0 relationship demonstrates that certain iconic prosodic effects can emerge when they align with existing physiological properties and do not conflict with core linguistic functions. The head angle-*f*0 mapping operates through biomechanical coupling that occurs naturally during spatial attention, creating a foundation for iconic prosody that potentially can be applied across languages. However, the absence of object size effects on lip opening and formant dispersion illustrates the limits of iconic influence when it encounters established linguistic constraints. The lack of observed effects challenges some assumptions about the scope of size-related iconic prosody and its interaction with segmental properties, suggesting that iconic prosody of size may be overshadowed by purely indexical functions. Articulatory parameters like lip opening and acoustic parameters like formant dispersion serve primary phonemic functions that take precedence over iconicity. The linguistic system appears to protect these core articulatory-acoustic relationships from interference, ensuring phonemic distinctiveness is maintained even when iconic pressures might favor different configurations.

This pattern suggests a hierarchical organization where iconicity operates within the boundaries set by linguistic necessity. *f*0 control, which serves multiple communicative functions beyond phonemic identity, remains more flexible and available for iconic expression. In contrast, articulatory and acoustic parameters that are tightly coupled to phonemic identity show greater resistance to iconic influence, particularly under conditions where phonemic requirements are salient. Iconic relations may therefore not be evident within a phoneme but across the vowel space, with high vowels being cross-modally associated with smallness and low vowels with larger size (Sapir, 1929). Our findings confirming H1 and rejecting H2 reveal how linguistic systems might channel iconic tendencies into specific domains while protecting others. Iconicity, as a core principle of language, is maintained without compromising other linguistic functions. From a practical standpoint, understanding these physiological mechanisms can inform fields such as speech synthesis, language teaching, and communication technologies. For instance, incorporating naturalistic head movement patterns into speech synthesis algorithms could enhance perceived naturalness and expressiveness. In language teaching, awareness of the sensorimotor aspects of speech production might aid in developing techniques for teaching prosody and intonation.

### Limitations

Several limitations of our study should be acknowledged. First, the exclusive inclusion of female participants, while controlling for gender-related differences in *f*0, limits the generalizability of our findings. Male voices, with inherently lower fundamental frequency, might exhibit different patterns in the relationship between head position and *f*0. However, we do not expect differences in this regard or are aware of studies that would make such predictions. Since *f*0 is to a large extent driven by social and cultural effects as well as gender identity, our results may be less constrained when analyzing only one biological sex. To exclude a potential male-female effect, future studies should include participants of all genders to explore potential differences and enhance the applicability of the results.

Second, our experimental paradigm involved specific task conditions that may have limited the expression of size-related iconic effects. Our study involved visual perception without physical interaction, whereas studies reporting size-related articulatory effects (Vainio, Mustonen, et al., 2019; Vainio et al., 2017; Vainio, Vainio, et al., 2019) involved direct manipulation of objects through grasping or their visualization. The haptic feedback and proprioceptive information available during physical object manipulation could provide more salient size cues in this specific task that translate into articulatory adjustments through stronger sensorimotor coupling than in our study where participants held a laser pointer but did not grasp or manipulate the object.

Third, the experimental setup, although designed to mimic a naturalistic task, through the limited vertical space, may have inadvertently constrained participants’ movements. The lack of a communicative intent, the laboratory environment, and the awareness of being observed could have led to more restrained head and lip movements than would occur in everyday speech or more interactive settings. However, we did not provide specific instructions regarding head movements or which parts of the arm and hand participants should move, allowing them to perform the task using their natural motion. This approach increases ecological validity, as it reflects how people gesture in real-world settings, and it introduces variability in movement strategies across participants. That said, large head rotations are probably less likely in general, as they have an impact on a complex motor system and affect posture and balance (Paloski et al., 2006).

We also attempted to mitigate these limitations by designing a task that encouraged natural movement and speech, using a game-like scenario to engage participants and make the task purposeful. The randomization of stimuli and the inclusion of familiarization trials were intended to reduce potential biases and learning effects. Our statistical models accounted for individual variability by including random intercepts and slopes, enhancing the robustness of our findings despite the controlled laboratory conditions.

Finally, the lack of a size effect may also be due to limitations in the way size was manipulated in our experiment. As illustrated in Figure 1, the can was enlarged proportionally in both height and width. However, in real life, cans often vary in height while their width remains constant. Our uniform scaling across two dimensions may have affected how natural the stimuli appeared to participants and may have limited their sensitivity to size differences. Consequently, the ability to detect effects of size on speech parameters could have been compromised.

## CONCLUSION

Our study highlights the role of vertical head position in modulating fundamental frequency, providing evidence for a physiological basis of iconic prosody. The robust effect of head rotation angle on *f*0 supports the notion that physical actions are intertwined with vocal expression, contributing to the body-entangled nature of language. Conversely, the anticipated effects of object size on lip opening and formant dispersion were not observed, suggesting that the relationship between object size and speech production may rather be indexical of vowel quality and speaker size than iconic of referent size.

These findings contribute to an understanding of how physiological and sensorimotor processes influence speech production and iconicity. They underscore the importance of considering physiological mechanisms in linguistic research and exploring the connection between body and language.

## Acknowledgments

The authors wish to thank Jörg Dreyer for the technical support. We are grateful to Cornelia Ebert and Manfred Krifka for their collaboration with the project, and Bodo Winter for the invaluable comments on an earlier version of this manuscript. We would also like to thank the reviewers whose comments encouraged us to improve the quality of this article. We thank the participants who took part in the study. A preliminary version of this manuscript was presented at CogSci 2019 and published in the proceedings. The submission is a reanalysis of a part of the first author’s dissertation.

## Funding Information

This work was supported by the German Research Foundation, grant number FU 791/6-1, project number 367155512 (in years 2018–2021) and CW 10/1-1, project number 502013782 (in years 2022–2025).

## Author Contributions

Both authors designed the study and collected the data. A.Ć. analyzed the data. A.Ć. wrote the first draft, both authors revised the manuscript.

## Ethics Approval

The ethical approval has been granted to the project by the German Society of Linguistics Ethics Commission with the number #2018-02-180912.

## Data Availability Statement

All anonymized, non-identifiable data, previous as well as current analyses, and models are available in the OSF repository: https://osf.io/e3cb4/.

## Note

^1^ A few examples are: Beyonce performing Listen, live at Oprah (https://youtu.be/pai1C2dsd3M, 2:05–2:20, 2:44–3:15), Demi Lovato’s performance of Stone Cold, live at Billboard 2015 https://youtu.be/B5qULV6x_cE, 1:05–1:12, 1:48–1:52, 2:18–2:24, 2:38–2:46), or Tiffany Mosley’s Total Praise, where she reaches notes as high as C#6 (https://youtu.be/UW7tfjP2Eho, 1:17–1:23, 1:58–2:01, 2:55–3:03, 4:45–5:10). All videos were last accessed on May 5, 2025.
